# The deviation of growth model for transparent conductive graphene

**DOI:** 10.1186/1556-276X-9-581

**Published:** 2014-10-20

**Authors:** Shih-Hao Chan, Jia-Wei Chen, Hung-Pin Chen, Hung-Sen Wei, Meng-Chi Li, Sheng-Hui Chen, Cheng-Chung Lee, Chien-Cheng Kuo

**Affiliations:** 1 Department of Optics and Photonics/Thin Film Technology Center, National Central University, 300 Chung-Da Rd, Chung-Li 32001, Taiwan; 2 Graduate Institute of Energy Engineering/Thin Film Technology Center, National Central University, 300 Chung-Da Rd, Chung-Li 32001, Taiwan; 3 Optical Sciences Center/Thin Film Technology Center, National Central University, 300 Chung-Da Rd, Chung-Li 32001, Taiwan

**Keywords:** Graphene, Transparent conductive electrode, Chemical vapor deposition

## Abstract

An approximate growth model was employed to predict the time required to grow a graphene film by chemical vapor deposition (CVD). Monolayer graphene films were synthesized on Cu foil at various hydrogen flow rates from 10 to 50 sccm. The sheet resistance of the graphene film was 310Ω/□ and the optical transmittance was 97.7%. The Raman intensity ratio of the G-peak to the 2D peak of the graphene film was as high as ~4 when the hydrogen flow rate was 30 sccm. The fitting curve obtained by the deviation equation of growth model closely matches the data. We believe that under the same conditions and with the same setup, the presented growth model can help manufacturers and academics to predict graphene growth time more accurately.

## Background

Graphene, comprising two-dimensional monolayer of sp^2^-bonded carbon atoms, has attracted substantial attention for use in transparent conductive electrodes (TCE), owing to its chemical stability and high optical transmittance from the ultraviolet to the infrared regions. Graphene as a TCE has a wide range of applications, including in solar cells, solid-state lighting, and detectors, owing to not only its higher optical transmittance but also its more favorable conductance [1-4] than those of traditional transparent conductive electrodes, such as indium tin oxide (ITO) and zinc oxide (ZnO) [5]. (Additionally, Furthermore, or Moreover), ITO is an expensive material, and it is unstable in chemical solution and cannot be utilized in a hydrogen-containing environment. ZnO-based thin film has also attracted interest for use in TCEs owing to its low cost, non-toxicity, and abundant constituent elements [6]. However, the properties of ZnO-based thin films are not uniform or stable. The several ways to produce graphene include mechanical exfoliation, graphene oxide (GO), and chemical vapor deposition (CVD). Mechanical exfoliation can yield high-quality graphene from graphite but this method produces graphene over a small area of the order of only a few tens of micrometers [4]. Graphene oxide can be formed by oxidizing graphite flakes; this method can produce large quantities of graphene whose electrical properties are, however, affected by the functional groups and various defects in the graphene [7]. Nevertheless, CVD is a promising method for growing high-quality graphene over a large area using Cu foils. Li et al. were the first to grow graphene over a large area of the order of square centimeters on Cu foil by CVD using methane, and this method has become a standard approach to forming graphene films in recent years [8]. Four-layered graphene exhibits a sheet resistance of about 350Ω/□, which represents a large step toward lower sheet resistance and a large increase in the range of graphene applications [9]. Additionally, various methods have been proposed to optimize the properties of CVD graphene [10-14]. This work develops a simply derived graphene growth model to predict the growth time with various hydrogen flow rates.

## Methods

Graphene films were grown by chemical vapor deposition (APCVD) on 25-μm-thick Cu foils (99.8%, Alfa-Aesar, item no. 13382) in a 3-in. quartz tube furnace under atmospheric pressure. Beforehand, electrochemical polishing (50% H_3_PO_4_ in deionized water of 100 mL) was utilized to smooth out the foil, and a voltage from 2 to 4 V was applied until the Cu foil glowed. Thereafter, the Cu foil was rinsed in a large amount of deionized water with sonication and then blow-dried with nitrogen gas. The Cu foil was placed in the reaction chamber, the Ar and H_2_ (1,000 and 2 sccm, respectively) gases were introduced into the chamber during temperature ramp-up. The Cu foil was annealed at 1,070°C for an hour. Then, 0.3 sccm of methane (purity, 99.99%) was used as a source of carbon to grow the graphene. The H_2_ flow rate was varied from 10 to 50 sccm prior to observation of the morphology of the graphene domains and the nucleation density. Following the growth process, the as-grown graphene/Cu foil was removed from the heating zone for rapid cooling. Polymethyl methacrylate (PMMA) was spin-coated on the as-grown graphene/Cu foil as a supporting layer to prevent any cracking during the transfer process. The graphene grew on both sides of the Cu foil. The graphene at the back of the foil was removed by floating on nitride acid solution (30% in deionized water) for 10 s. The Cu foil was etched away overnight using an ammonium persulfate solution (0.1 M) and then rinsed three times in deionized water. The PMMA/graphene was placed on the substrate, and the PMMA was then dissolved in hot acetone bath for 24 h. The residual PMMA was removed by annealing in air at 200°C for an hour and reduced to pristine graphene using an H_2_/Ar (7/20 sccm) mixture. The morphology and the nucleation density of the graphene domain were measured by scanning electron microscopy (SEM); The surface profile of Cu foils were measured by atomic force microscopy (AFM); the sheet resistance was measured using a four-probe stage; the Raman shift of the graphene was measured by Raman spectroscopy using a laser with a wavelength of 532 nm, the laser power at the focused spot was 2 mW, and the numerical aperture value was 0.75 on the sample with an area of 1 μm^2^.

## Results and discussion

**Figure 1:**
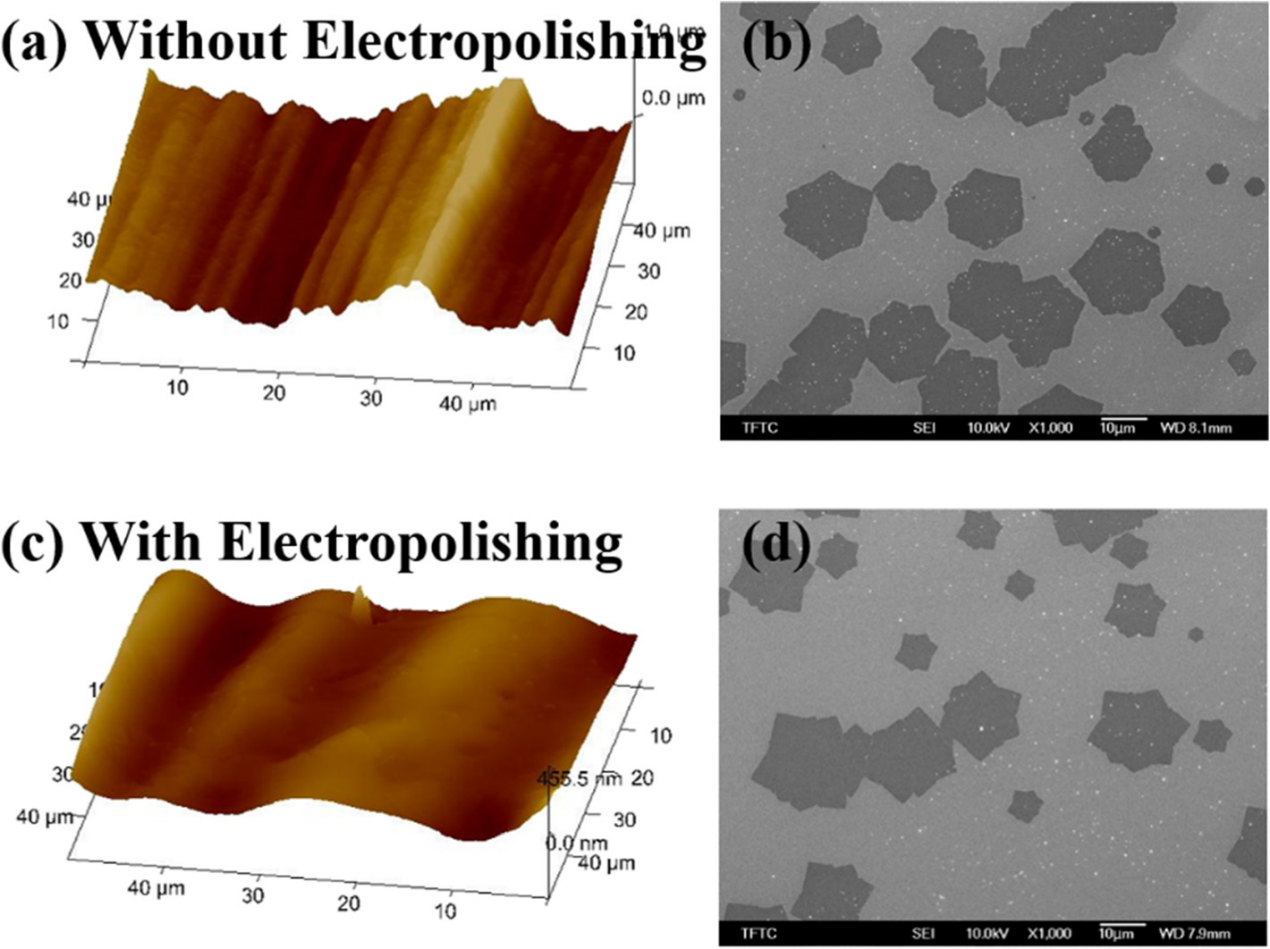
**Surface profile of Cu foil and SEM images of graphene domains.** Surface profile of Cu foil **(a)** before and **(c)** after electropolishing. **(b)** SEM image of morphology of graphene domains **(c)** before and **(d)** after electropolishing.

**Figure 2:**
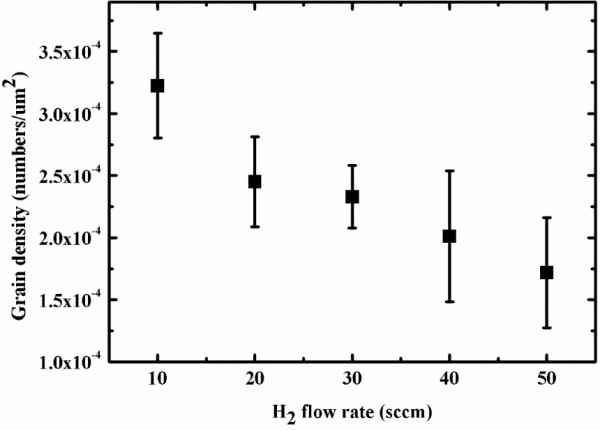
Calculation of graphene domain densities with various hydrogen flow rates from 10 to 50 sccm.

**Figure 3:**
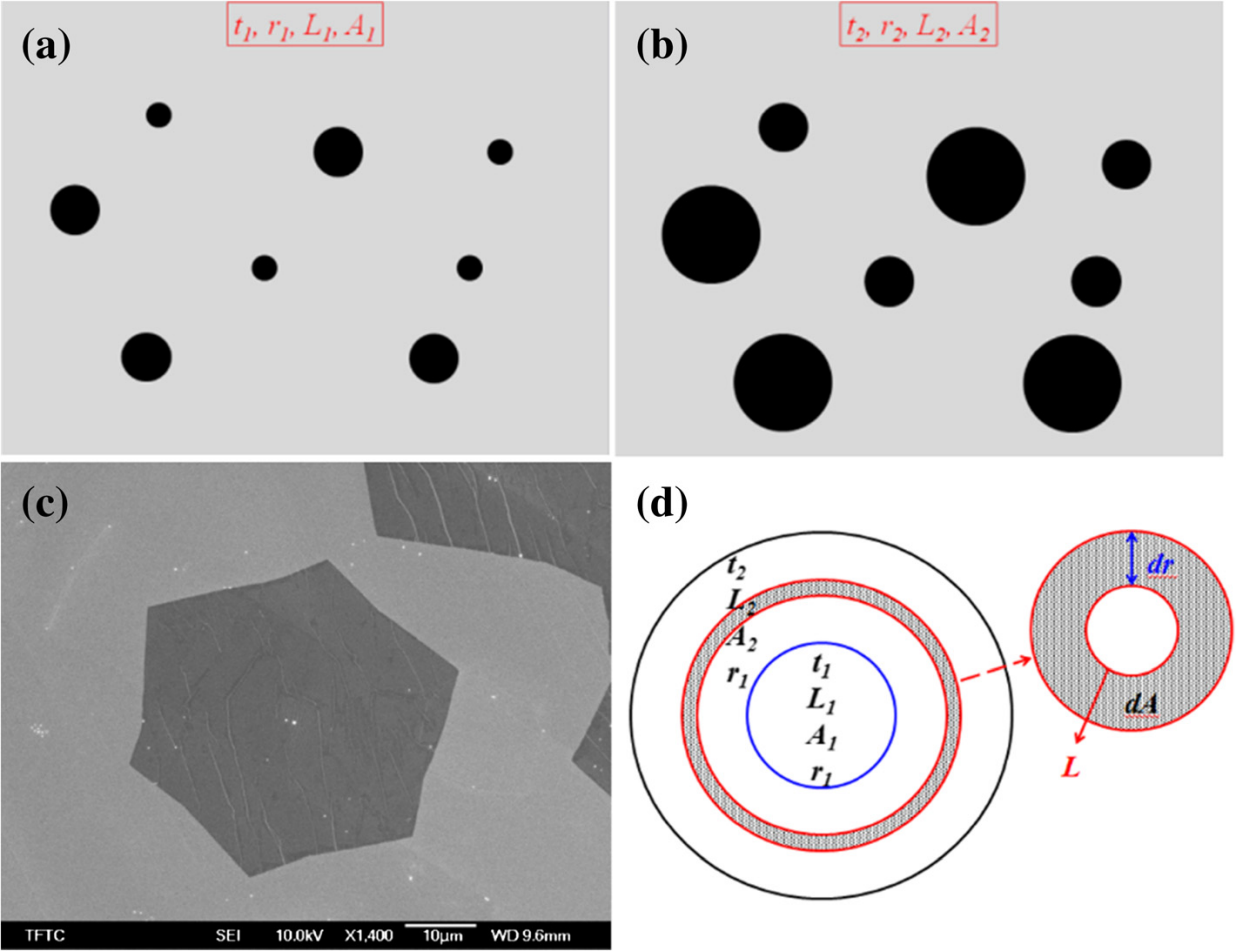
**Results of growth model and SEM image.** Results of growth model with growth time of **(a)** t_1_ and **(b)** t_2_. **(c)** SEM image of hexagonal graphene domain with hydrogen flow rate of 30 sccm*.***(d)** The deviation of growth model for graphene domain.

The number of graphene layers, the shape, and the nucleation density were significantly influenced by the surface roughness of Cu foil. Figure 1a,c presents AFM images of the reduction of the rolling marks on the Cu foil by electropolishing. The domain density of graphene clearly declined from Figure 1b-d, and the graphene morphology became star-shaped under hydrogen at a 1flow rate of 20 sccm. In the latter experiments, the electropolishing of Cu foils were applied for growing graphene films. To synthesize a larger graphene domain, experiments were conducted in which the hydrogen flow rate was increased from 10 to 50 sccm, and the graphene domain density is calculated, as displayed in Figure 2. A previous investigation revealed that the hydrogen flow rate importantly affects the graphene growth mechanism, owing to the etch effect and its effect on the graphene domain density. The density of graphene nuclei is reduced as the hydrogen flow rate is increased. The hydrogen flow rate also affects the morphology of graphene. In this case, the graphene had a hexagonal shape when the hydrogen flow rate was 30 sccm, as shown in Figure 3c. A growth model to elucidate the rate of graphene domain growth was proposed. If the graphene domain is circular, then this model allows the easy calculation of the difference between the graphene domain sizes at two growth times. Figure 3 displays the derivation of the growth model. Figure 3a,b schematically depicts the growth of the graphene domains from growth time *t*_*1*_ to *t*_*2*_. Figure 3d presents the growth model in detail, where *L* is the circumference of graphene domain; *A* is the mean area of the grown domains, and the *r* is the average radius of the domains. Now, an area factor is sought such that

(1) dA=Ldr

For a carbon deposition rate *v*, the deposition rate of carbon atoms at one direction is given by,

(2) $$v=\frac{dr}{dt}$$

Combining Equations 1 and 2 yields simultaneous equations,

(3) $$\left\{\begin{array}{c} dA=\mathrm{Lvdt}\\ L={\left(4\pi A\right)}^{\frac{1}{2}} \end{array}\right)$$

Solving Equation 3 yields,

(4) $${\left(4\pi A\right)}^{-\frac{1}{2}}dA=vdt$$

(5) $$\frac{1}{2\pi }{\left(4\pi A\right)}^{\frac{1}{2}}=vt$$

(6) $$A=\pi {\left(vt\right)}^{2}$$

The mean area *A* now becomes

(7) $$A=\pi {\left(vt\right)}^{2}=\frac{1}{2}\frac{d^{2}A}{dt^{2}}t^{2}$$

**Figure 4:**
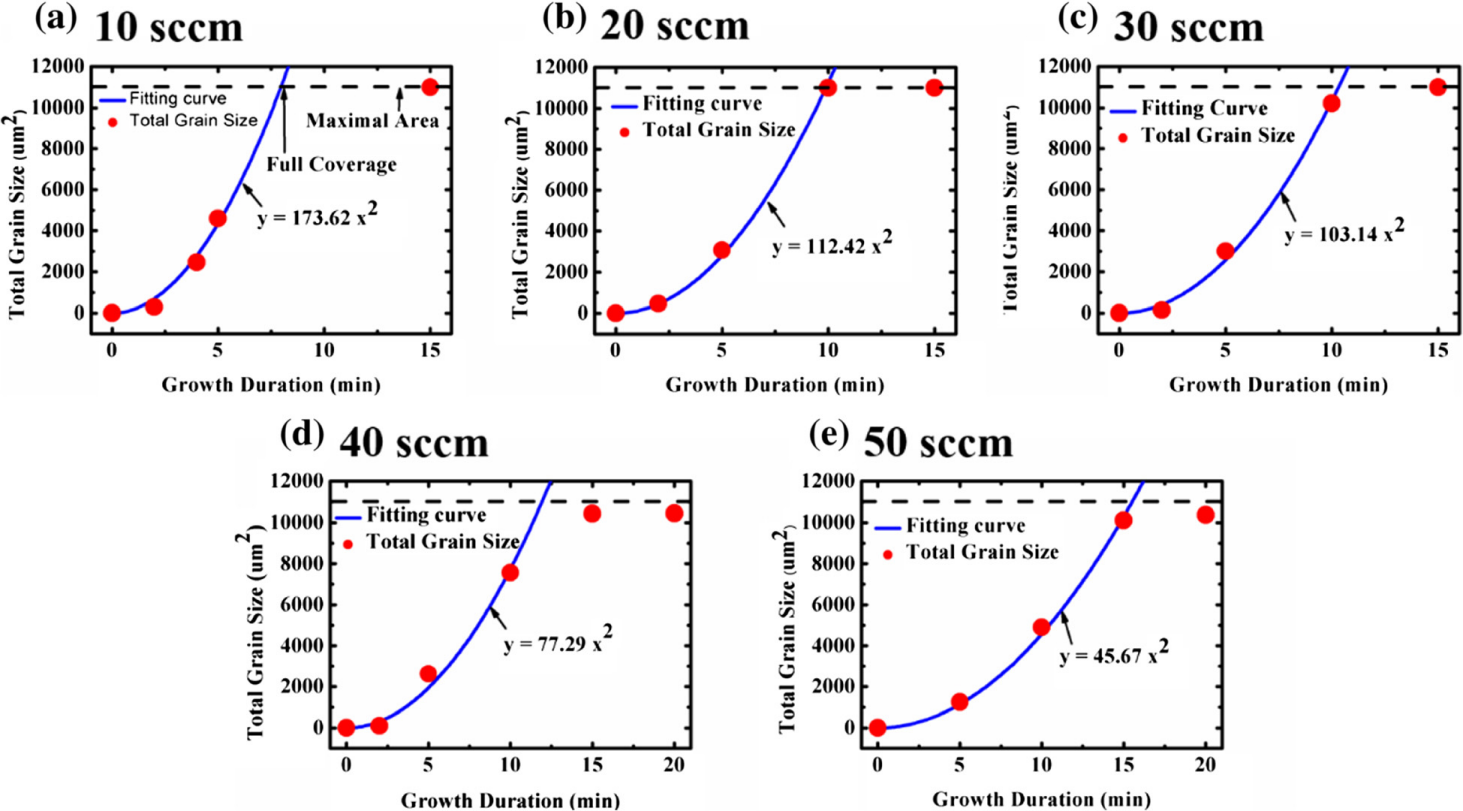
**Growth model of graphene at various hydrogen flow rates and corresponding fitting curves. (a)** 10, **(b)** 20, **(c)** 30, **(d)** 40, and **(e)** 50 sccm.

**Figure 5:**
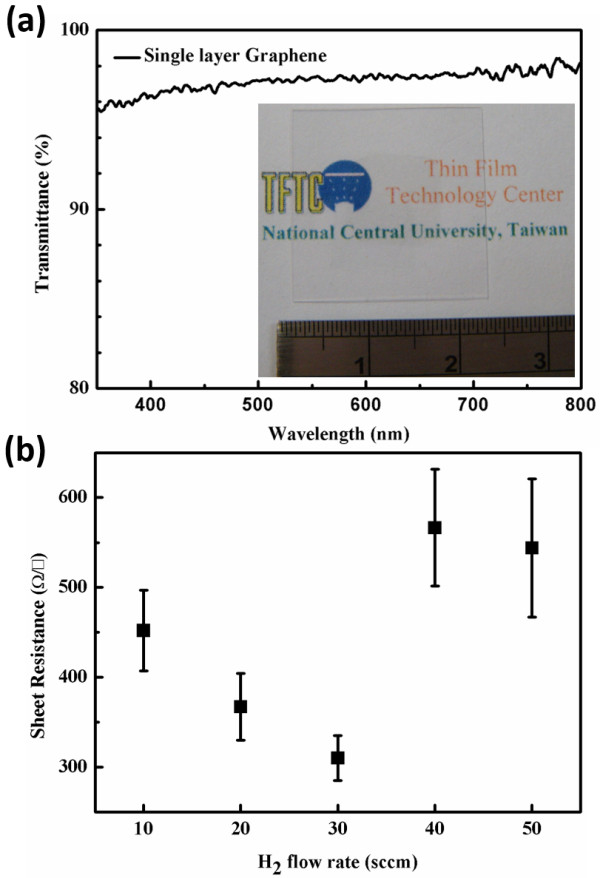
**Optical transmittance and sheet resistance of graphene film. (a)** Optical transmittance of graphene film. **(b)** Sheet resistance of graphene film obtained using various hydrogen flow rates from 10 to 50 sccm.

**Figure 6:**
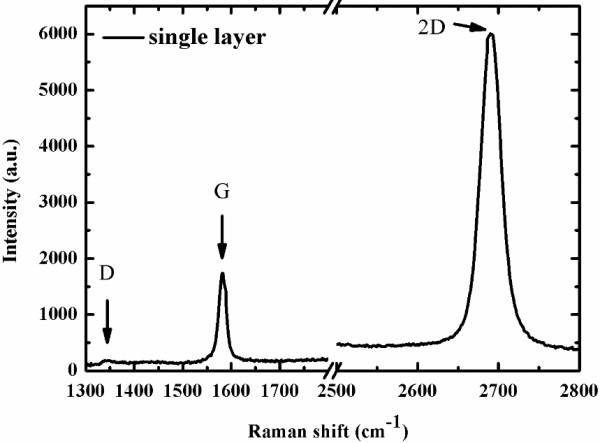
Raman spectrum of single-layer graphene film.

The *A* is a quadratic equation which means the graphene domains grown with an acceleration rate on the Cu foil. Equation 7 can be utilized to predict the coverage area of graphene on the Cu foil under various hydrogen flow rates from 10 to 50 sccm, as displayed in Figure 4. The dashed line is the area of the Cu foil (1.1 cm^2^) and the red spots represent the growth times of graphene that yield the specified area, based on Equation 7. A red spot at the dashed line indicates that the graphene fully covered the Cu foil. Figure 4 reveals that the graphene fully covered the foil when the hydrogen flow rate was 10, 20, or 30. When the hydrogen flow rate exceeded 30 sccm, the graphene did not fully cover the Cu foil because of the etching effect and thermal equilibrium occurs on the edge of graphene domains. Also, a fitting equation is obtained for the growth of graphene with different hydrogen flow rate, which is plotted as the blue curve. Based on the developed growth model, we can adjust any coverage of graphene on the Cu foil which closely matches the fitting curve; the growth model predicts the growth rate of graphene. As mentioned above, the graphene domain was hexagonal when the hydrogen flow rate was 30 sccm. The graphene fully covered the Cu foil both according to the growth model and in the experiment. The graphene was transferred onto a glass substrate to measure its transparency, sheet resistance, and Raman shift. In Figure 5a, the graphene film is placed on the glass substrate with a relatively high transmittance of about 97.7% at λ =550 nm. The attenuation coefficient (α =2.3%) was fitted using Beer’s law; the value matches the theoretical value of 2.3% when the λ =550 nm [15]. The graphene films that were synthesized using various hydrogen flow rates were also transferred to the substrate and their sheet resistance was measured, as shown in Figure 5b. The lowest obtained sheet resistance of graphene was 310Ω/□, which was achieved when the hydrogen flow rate was 30 sccm, because the smooth edge of the graphene domain reduced the number of scattering centers which inhibited the carrier transportation. The sheet resistance increased with the hydrogen flow rate over 30 sccm because the graphene did not fully cover the Cu foil, and because the larger number of pores in the graphene increased the sheet resistance. Figure 6 shows the Raman spectrum of graphene film, in which the peaks are typical of a single-layer graphene, with a 2D/G ratio of as high as ~4 when the hydrogen flow rate was 30 sccm. The full width at half maximum (FWHM) was 23.5 cm^-1^, verifying the presence of a single-layer graphene [8].

## Conclusions

An approximate growth model of the synthesis of graphene by APCVD was developed. When the hydrogen flow rate was 30 sccm, the transmittance as a function of wavelength for single-layer graphene reached its maximum of 97.7% at λ =550 nm. The 2D/G ratio and the FWHM indicated that the graphene comprised a single layer of high quality. The lowest obtained sheet resistance of a single layer of graphene was about 310Ω/□. The results of the experiments closely matched the fitting curve. We believe that, under the same conditions and with the same experimental setup, the proposed growth model can help manufacturers and academics predict the growth time of graphene more accurately.

## Competing interests

The authors declare that they have no competing interests.

## Authors’ contributions

SHC (Chan) designed the study and wrote the paper. JWC, HSW, MCL, and HBC analyzed the data. SHC (Chen), CCL, and CCK are advisors. All authors read and approved the final manuscript.
